# Intracellular Neural Recording with Pure Carbon Nanotube Probes

**DOI:** 10.1371/journal.pone.0065715

**Published:** 2013-06-19

**Authors:** Inho Yoon, Kosuke Hamaguchi, Ivan V. Borzenets, Gleb Finkelstein, Richard Mooney, Bruce R. Donald

**Affiliations:** 1 Department of Electrical and Computer Engineering, Duke University, Durham, North Carolina, United States of America; 2 Department of Neurobiology, Duke University Medical Center, Durham, North Carolina, United States of America; 3 Department of Physics, Duke University, Durham, North Carolina, United States of America; 4 Department of Computer Science, Duke University, Durham, North Carolina, United States of America; 5 Department of Biochemistry, Duke University Medical Center, Durham, North Carolina, United States of America; University of Antwerp, Belgium

## Abstract

The computational complexity of the brain depends in part on a neuron’s capacity to integrate electrochemical information from vast numbers of synaptic inputs. The measurements of synaptic activity that are crucial for mechanistic understanding of brain function are also challenging, because they require intracellular recording methods to detect and resolve millivolt- scale synaptic potentials. Although glass electrodes are widely used for intracellular recordings, novel electrodes with superior mechanical and electrical properties are desirable, because they could extend intracellular recording methods to challenging environments, including long term recordings in freely behaving animals. Carbon nanotubes (CNTs) can theoretically deliver this advance, but the difficulty of assembling CNTs has limited their application to a coating layer or assembly on a planar substrate, resulting in electrodes that are more suitable for *in vivo* extracellular recording or extracellular recording from isolated cells. Here we show that a novel, yet remarkably simple, millimeter-long electrode with a sub-micron tip, fabricated from self-entangled pure CNTs can be used to obtain intracellular and extracellular recordings from vertebrate neurons *in vitro* and *in vivo*. This fabrication technology provides a new method for assembling intracellular electrodes from CNTs, affording a promising opportunity to harness nanotechnology for neuroscience applications.

## Introduction

Intracellular electrodes fashioned from glass pipettes have been available for decades, but their fragility and high impedance motivates the search for novel probes with improved electrical and mechanical properties [1]–[18]. Indeed, diverse approaches have been used to improve recording electrodes, including new coating layers [1], [4], substrate-dependent platforms [2], [3], [5]–[10], new fabrication methods for metal electrodes [11], and incorporation of new materials [12]–[17]. Three requirements that any novel intracellular recording technology must meet are electrochemical stability, resistance to bio-fouling, and mechanical compatibility with brain tissue. Carbon nanotubes (CNTs) display high electrical conductance, pronounced mechanical strength, and large surface area, which along with their demonstrated electrochemical and biological stability [18], indicate that they have the potential to meet these requirements. However, the difficulty of assembling CNTs has restricted their use to surface coating over a flat substrate or wires [1]–[3], limiting their previous application to *in vivo* extracellular recording from a cortex [1], stimulation on a separated muscle [2], or extracellular recording from isolated retinas [3].

An intracellular electrode made out of pure CNTs could exploit the attractive electromechanical properties of this material but requires a relatively long (∼ 1 mm) insulated shaft to penetrate into brain tissue and an exposed tip of sub-micron diameter to impale and stably record from neuronal cell bodies, which are 5–50 µm in diameter in the vertebrate brain. With this goal in mind, we developed a procedure involving dielectrophoresis, annealing, insulation coating, and tip exposure to make a self-entangled, needle-shaped CNT probe suitable for obtaining intracellular recordings from vertebrate neurons.

## Materials and Methods

### Dielectrophoresis

**Figure 1:**
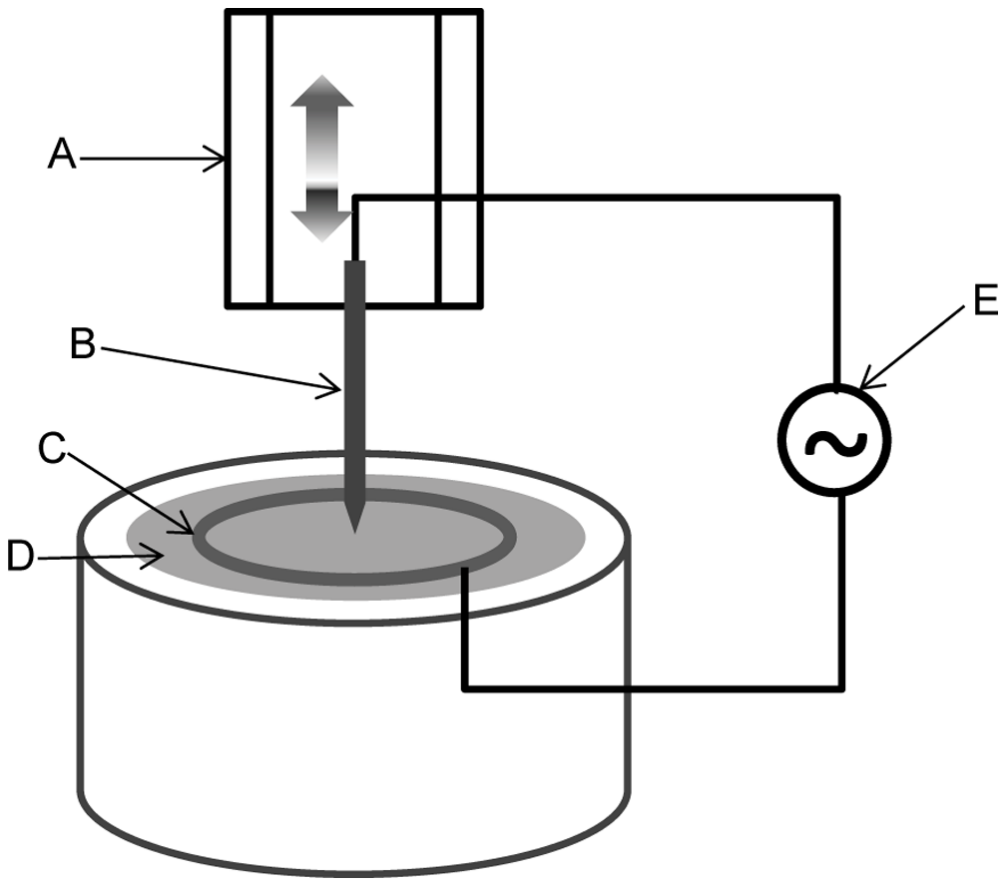
CNT fibril dielectrophoresis “pulling” stage assembled for this study. (A) A motorized linear stage moves only in the vertical direction, pulling the tungsten wire out of the solution. (B) A electrochemically sharpened tungsten tip functioned as a source electrode. (C) A submerged metal ring functioned as a counter electrode. (D) CNT dispersed solution. (E) High-frequency AC power source.

The self-entangled MWCNT probe was made by dielectrophoresis with an electrochemically sharpened tungsten wire (diameter 125 µm) and MWCNT dispersed in solution. The electrochemical etching process was described previously [19]. MWCNTs (outer diameter 8–15 nm, 95 wt%) were purchased from Cheap Tubes. The solution was prepared by 3 steps: mixing, sonication, and centrifugation. MWCNT 0.4 g, Polyvinylpyrrolidone (PVP, surfactant) 0.12 g, and deionized water (DIW) 40 ml were mixed and sonicated with a high-intensity probe type ultrasonic processor ([(30 sec maximum amplitude +10 sec pause) × 10 times] repeated 1 to 3 more times with ice cooling its container in between). Non-dispersed MWCNTs were precipitated by centrifuge (3,000 RPM, 20 minutes) and then discarded. The dielectrophoresis process [20], [21] used an electrochemically etched tungsten wire as the source electrode and a 25 mm diameter metal ring submerged beneath the surface of the MWCNT dispersed solution as a counter electrode. The sharp tip of the tungsten wire was placed to touch the solution in the middle of the counter electrode (see Figure 1). The tungsten wire and the counter electrode were then electrically connected to a power source, which supplied a sinusoidal 10 MHz signal, 40–80 V peak to peak amplitude. The tungsten wire was slowly pulled (40 µm/sec) out of the solution. The pulling speed was increased toward the end for growth termination at a desired length and to create a tapered end.

### Annealing

**Figure 2:**
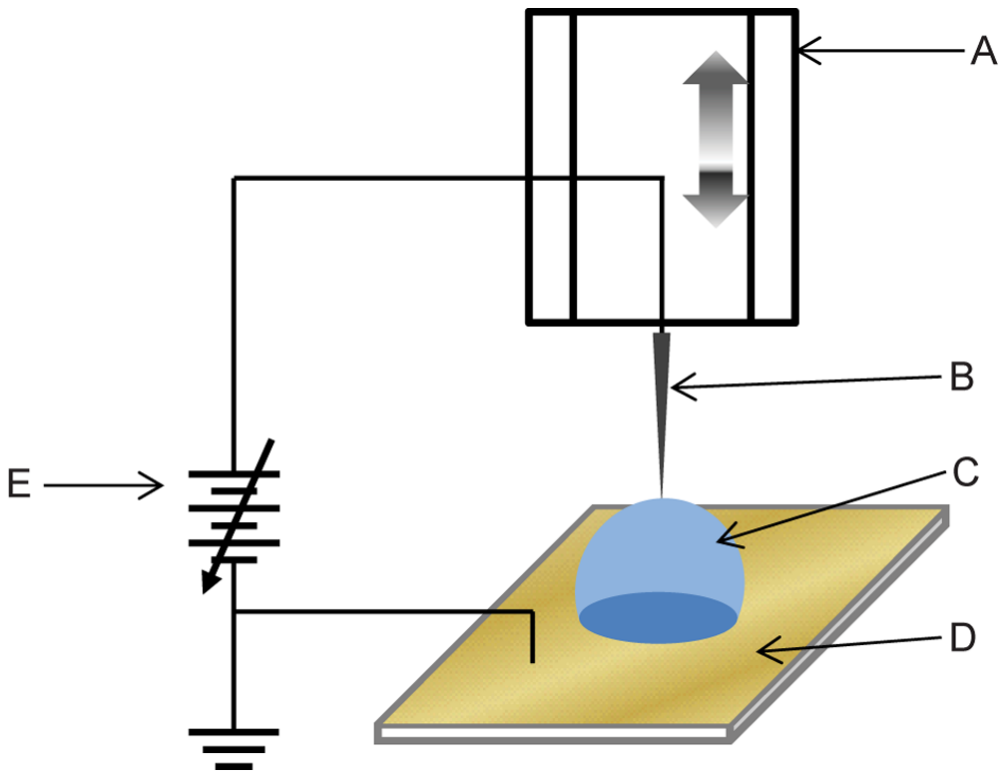
CNT fibril annealing setup. (A) A motorized linear stage moving only in the vertical direction. (B) A CNT probe. (C) Water droplet. (D) Gold plated surface for grounding. (E) Variable DC voltage source.

Using a micro-stage while monitoring the proximity with an optical microscope, the end of the CNT probe was placed to touch the top of a water droplet on a grounded surface (see Figure 2). DC voltage applied to the probe was ramped up to a threshold value around 80 V with a limited current by a 10 MΩ. When a threshold voltage was reached, a few microns of the probe tip got cut-off generating tiny water mist nearby.

### Parylene-C Coating and FIB (Focused Ion Beam) Tip Exposure

LPPVD (Low-Pressure Physical Vapor Deposition, Cookson Electronics PDS 2010 LABCOTER2) was used in-house for the coating. Around 250 nm thickness of Parylene-C was coated homogeneously in the deposition chamber where the CNT probes were hung downward in the middle of the chamber. For the tip exposure, FEI Quanta 200 3D FEG system was used. Ga^+^ beam was accelerated to 30 kV and its current was 1 nA for rough cutting and 100 pA for final trimming.

### Cyclic Voltammetry and Impedance Spectroscopy

A three-electrode electrochemical cell was used with an impedance analyzer setup (ECI (Electrochemical Interface) and FRA (Frequency Response Analyzer) from Solatron Analytical). An AgCl/Ag pellet was used as the reference electrode, a metal mesh as the counter electrode, and 25 mM PBS (Phosphate buffered saline) is used to mimic the osmolarity of the brains. During these measurements, controlling the contact area between the CNT probe and phosphate buffered saline (PBS) is challenging, because wetting area of the solution on the CNR probe is not linearly proportional to dipping depth. Therefore different dipping depth into the PBS solution gives a range of measurement for any sample. Given that, both CV and EIS gave reliably repeatable results for each single sample (see Appendix S1 and Figure S1 for further details).

### Intracellular Recording

Mice (*Mus musculus*, 34 days post natal, VGAT-ChR2-YFP line 8 (The Jackson Laboratory Stock Number: 014548, VGAT stands for vesicular glutamic acid transporter, ChR2 for channelrhodopsin 2, and YFP for Yellow Fluorescent Protein.) were used for the intracellular recording, in accordance with a protocol approved by the Duke University Institutional Animal Care and Use Committee under protocol A292-11-11. After induction of inhalation anesthesia (Isoflurane), the mouse was decapitated, and the brain was removed rapidly and placed in oxygenated ice-cold Ca-free artificial CSF (ACSF). Coronal brain slices were cut at 400 µm thickness and transferred to a holding chamber (room temperature) for 2–4 h. Individual slices were transferred to an interface-type chamber (30°C; Medical Systems) for intracellular recordings. The ACSF consisted of (in mM) 119 NaCl, 2.5 KCl, 1.3 MgCl_2_, 2.5 CaCl_2_, 1 NaH_2_PO_4_, 26.4 NaHCO_3_, and 11 glucose, equilibrated with 95% O_2/_5% CO_2_. Intracellular potentials were passed through a voltage follower (HS-2A ×0.1 L, Axon Instruments, leakage current 1 pA), low-pass filtered at 10 kHz, then digitized at 10 kHz. Targeted brain areas were cortex layer II/III or V. For an electrical stimulation, a concentric bipolar electrode (which looks like a blunt needle) was placed near recording site on the brain slice, and injected positive or negative step function current (typically 10 µA) briefly (1–10 ms). For the optical stimulation, an optical fiber was pointed directly above the recording site to deliver blue laser light (wavelength = 473 nm).

### Extracellular Recording

All experiments were performed in accordance with protocol approved by the Duke University Institutional Animal Care and Use Committee under protocol A292-11-11. Male mice (*Mus musculus, ∼*30 days old) were anesthetized with ketamine (46 mg/kg) and xylazine (24 mg/kg) and supplemented with isoflurane (0.5–1.5%) in oxygen. Core temperature was monitored with a rectal probe (BAT-12, Physitemp Instruments) and maintained at 36±1°C with a heating pad. A midline incision of the scalp was performed, and the skin was retracted. A small metal pin was attached to the cranium with dental cement, and the animal was fixed in a custom-made stereotaxic stage. A small craniotomy (∼200 µm^2^) was performed over somatosensory cortex, and the dura overlying the brain was resected. The cortical surface was kept continuously moist. The electrode was lowered into the brain 100 - 1,000 µm with a hydraulic micromanipulator (SD Instruments). Signals were amplified 1000 times and band pass filtered (100 Hz –10 kHz) with a differential amplifier (A-M Systems) before being recorded with Spike 2 (Cambridge Electronic Design) at a sampling rate of 10 kHz.

## Results

**Figure 3:**
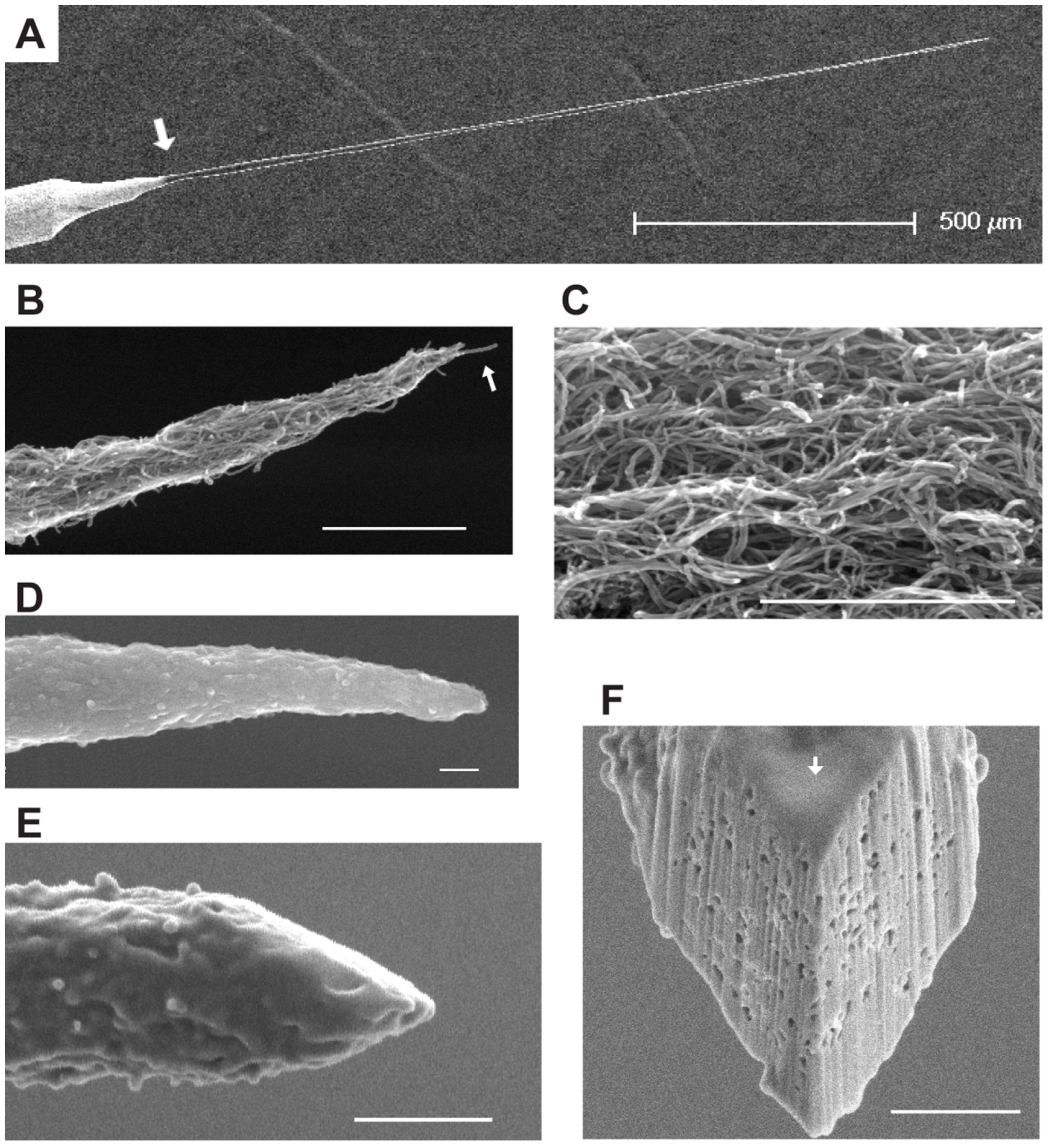
SEM images of CNT probes. (A) Low magnification view of a CNT probe. Tungsten wire extends from lower left to the white arrow; CNT probe extends from the arrow for 1.5 mm to upper right. (B) Tip of a CNT probe after dielectrophoresis. The tip tapers down to a single CNT (white arrow). (C) CNTs in the probe show a clearly self-entangled morphology. (D) A CNT probe tip after coating with 300 nm Parylene-C, which homogeneously covers the entire probe. (E) An exposed CNT probe tip after FIB cutting. Two FIB cutting planes are perpendicular to the picture, crossing at the end of the probe. (F) An angled view (at 40° with respect to the electron beam in the SEM) of exposed CNT probe tip shows the two cut planes. The FIB cutting did not damage nearby insulation coating, which is clearly visible (white arrow). Scale bars in (B)- (F): 1 µm.

The tip of an electrochemically sharpened tungsten wire served as the foundation from which a pure CNT probe was elaborated. We optimized the process to draw untapered probes of ∼ 1 mm length and 5–10 µm diameter from a multi-walled CNT (MWCNT) solution. In the dielectrophoresis process, important parameters were: applied potential, speed of pulling, and concentration of dispersed CNTs in the dielectrophoresis solution. We found the diameter of CNT probes was proportional to amplitude of the applied potential and concentration of CNTs in the solution, and inversely to the speed of pulling. The combination of parameters was optimized for homogeneity of diameter along the length of the CNT probe. Finally, the tapering toward the last 100 µm was achieved by increased pulling speed. An annealing step stabilized the electrochemical performance of the CNT probe and improved its mechanical stiffness, but did not significantly alter its impedance characteristics. A 300 nm thick Parylene-C coating was applied along the length of the probe’s shaft (Figure 3D) and a focused ion beam (FIB) was used to expose and shape the conductive CNT probe tip in an angled shape so that it resembled the blade of a flat-head screw driver (Figure 3E,F).

**Figure 4:**
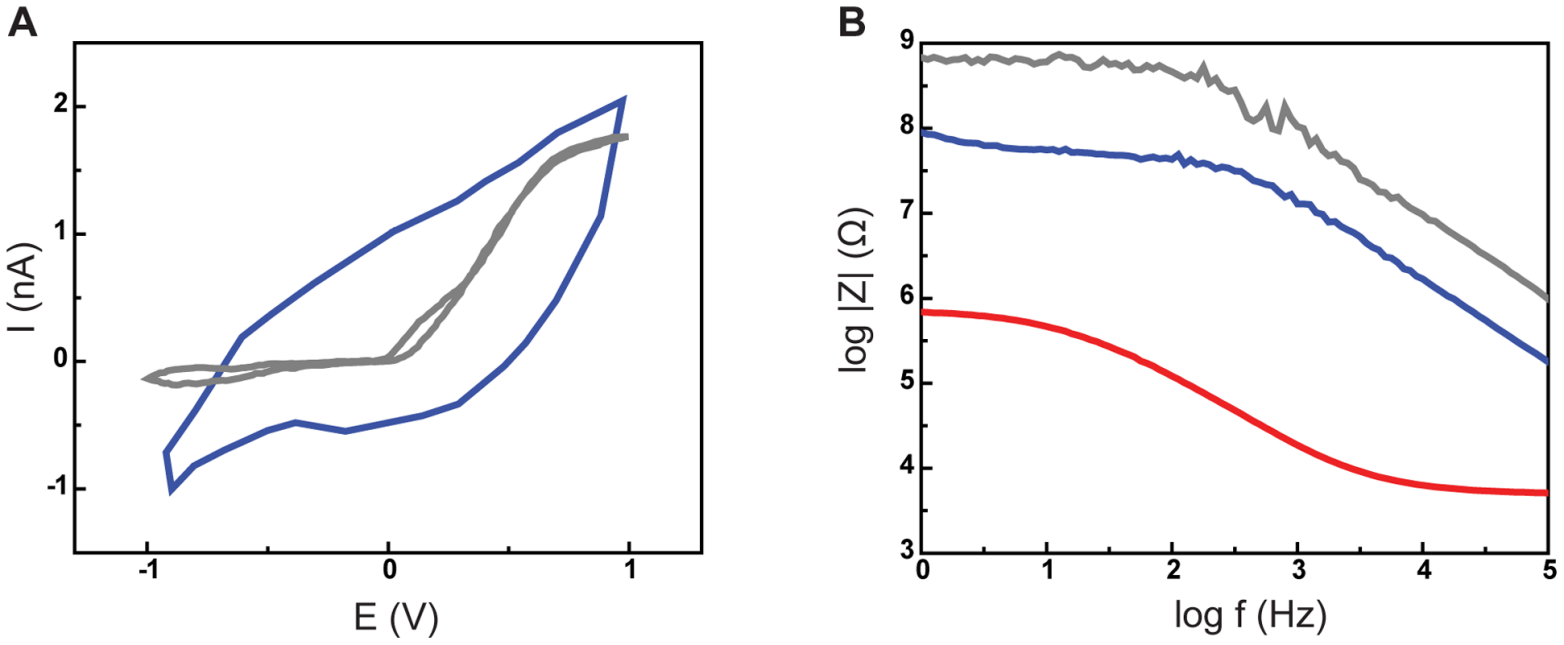
Impedance characterization of CNT probes. (A) Cyclic Voltammetry (CV) shows the CNT probes (with the insulation and FIB cut) have significantly larger charge transfer (blue line), compared to the sharp intracellular-recording glass pipette (gray line). (B) Electrochemical impedance spectroscopy (EIS) shows significantly lower impedance of the CNT probe (blue line) compared to the glass pipette (gray line) over the frequency range of 1 to 100 kHz. Successful insulation-coating plus FIB-cutting reduces contact area between the CNT probe and solution by a few orders of magnitude, which explains the difference between the curves measured before coating (red line) and after FIB cutting (blue line).

The electrochemical characteristics of the CNT probes (*n* = 5) were measured by cyclic voltammetry (CV) and electrochemical impedance spectroscopy (EIS). As Zheng et al. described extensively [22], a classical simple interface model is not applicable for an ionic liquid and CNT electrode interface. However, we can obtain the following qualitative information and comparisons from the measurements. There are no oxidation or reduction peaks in CV (Figure 4A) within the applied potentials ranging from −1 to +1 V, which indicates sufficient electrochemical inertness of the CNT probe. From the EIS measurement (Figure 4B), we can deduce the quality of insulation on the CNT probes and the recording signal quality. A properly applied insulation coating will increase the impedance by reducing contact area significantly, yet relatively low impedance (<100 MΩ) over typical operating frequencies (1 to 100 kHz) is desired, since it can improve signal quality. These data, coupled with SEM inspection of the CNT probe, imply that the increased impedance measured after Parylene-C coating and FIB cutting of the probe, is a direct result of our successfully reducing the contact area. Finally, impedance measurements revealed that the impedance of CNT probes (*n* = 5) were an order of magnitude lower than conventional glass electrodes (*n* = 3) over a frequency range of 1 to 100 kHz. These electrochemical features have two practical implications; (1) the lower impedance of the CNT probes can yield high signal to noise recordings. (2) the relatively large capacitance of the CNT probe (Figure 4A) is difficult to compensate during current injection state. Therefore, we used the CNT probes for passive voltage measurements only.

**Figure 5:**
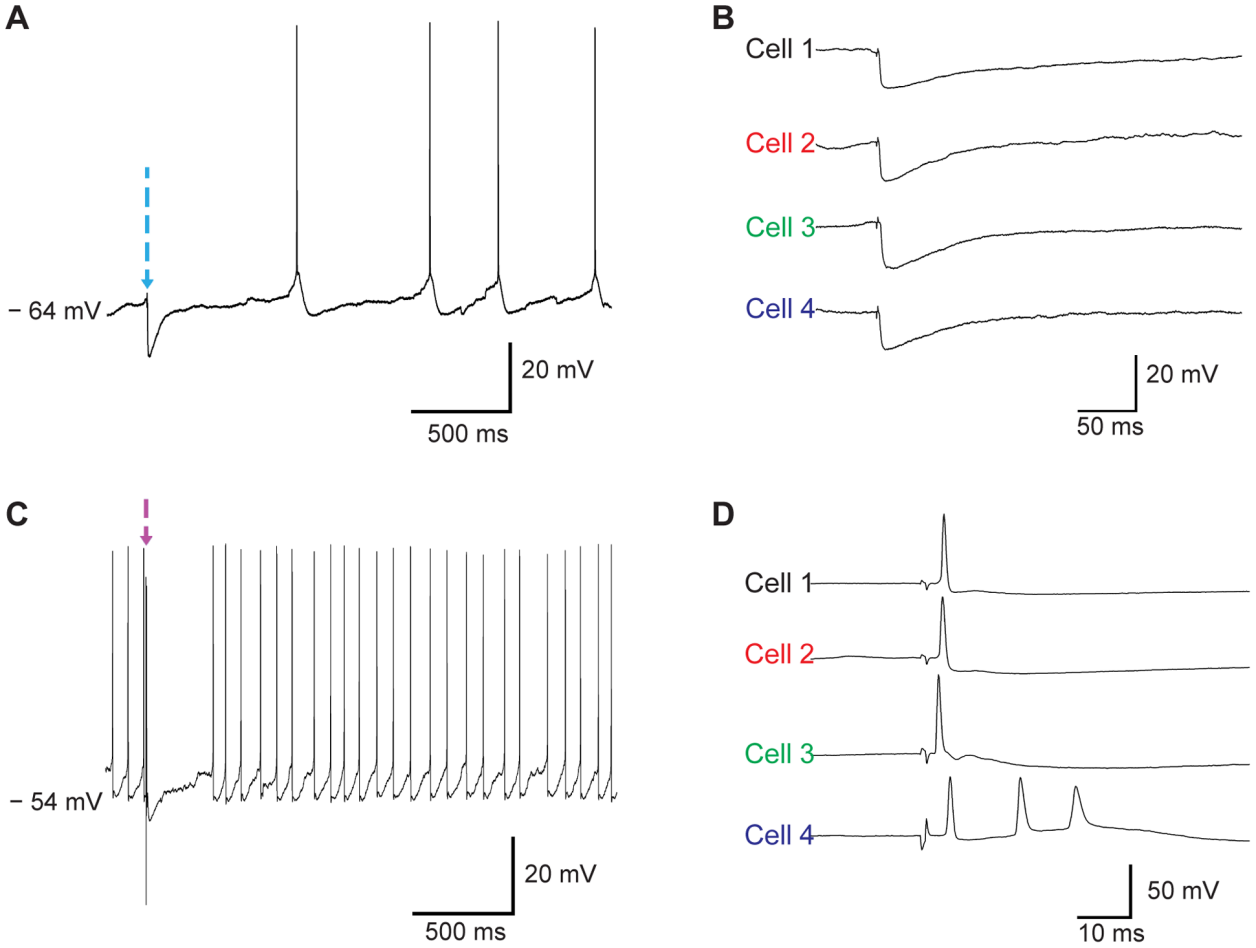
*In vitro* brain slice intracellular recording from mouse, cortical neurons. (A) Light-evoked inhibitory post synaptic potential (IPSP) recorded with a CNT probe from a neuron in cortical brain slice prepared from a VGAT-CHR2 mouse. A brief light pulse was applied at the time marked by the arrow (V_m_ = −64 mV). (B) Expanded membrane potential records for CHR2 evoked IPSPs collected from four different cortical neurons. (C) Electrically-evoked excitatory postsynaptic potential (EPSP) from a cortical neuron in a mouse cortical brain slice (V_m_ = −54 mV). (D) Expanded membrane potential records of electrically-evoked EPSPs collected from four different cortical neurons.

To begin to explore the suitability of the CNT probes for neural recording, we first attempted to obtain intracellular recordings from cortical neurons in brain slices prepared from transgenic mice in which the light-activated ion channel channelrhodopsin 2 (ChR2) [23]–[25] was placed under the promoter for the vesicular glutamic acid transporter (VGAT), thus restricting expression of ChR2 to GABA-releasing inhibitory neurons. When a ChR2 protein absorbs blue light, it forms a nonspecific cation channel. Therefore, blue light stimulation brings cations into ChR2-expressing neurons that depolarize the cell and lead to firing of action potential(s). Focal illumination of the brain slice in the region of the recording site was then used to activate inhibitory synapses on an impaled cell, while a concentric bipolar stimulation electrode placed adjacent to the recording site to electrically stimulate a population of neurons which will drive a mixture of excitatory and inhibitory synaptic inputs. Using this approach, we were able to make intracellular recordings from a total of four cells using three different CNT probes (out of 6 CNT probes), with a mean recording time of 3 minutes (198.1±41.7 seconds, Figure 5 and Figure S2). Successful penetration was accompanied by a sharp drop in membrane potential (−57±4.62 mV), and enabled the detection of spontaneous synaptic potentials and action potentials (61.53±7.92 mV, Time course of action potentials is shown in Figure S3). Focal illumination with 473 nm light pulses (2 ms) delivered via a 200 µm (core diameter) micron fiber optic evoked short latency (4.94±0.84 ms) hyperpolarizing membrane potential responses (11.86±1.43 mV) in the impaled cell, consistent with ChR2-mediated activation of inhibitory inputs. Electrical stimulation evoked short latency depolarizing responses accompanied by action potentials or biphasic responses consisting of depolarizing and hyperpolarizing components, consistent with electrical activation of excitatory and inhibitory inputs. These observations indicate that the CNT probe developed here is suitable for intracellular recordings of membrane potential from cortical neurons.

**Figure 6:**
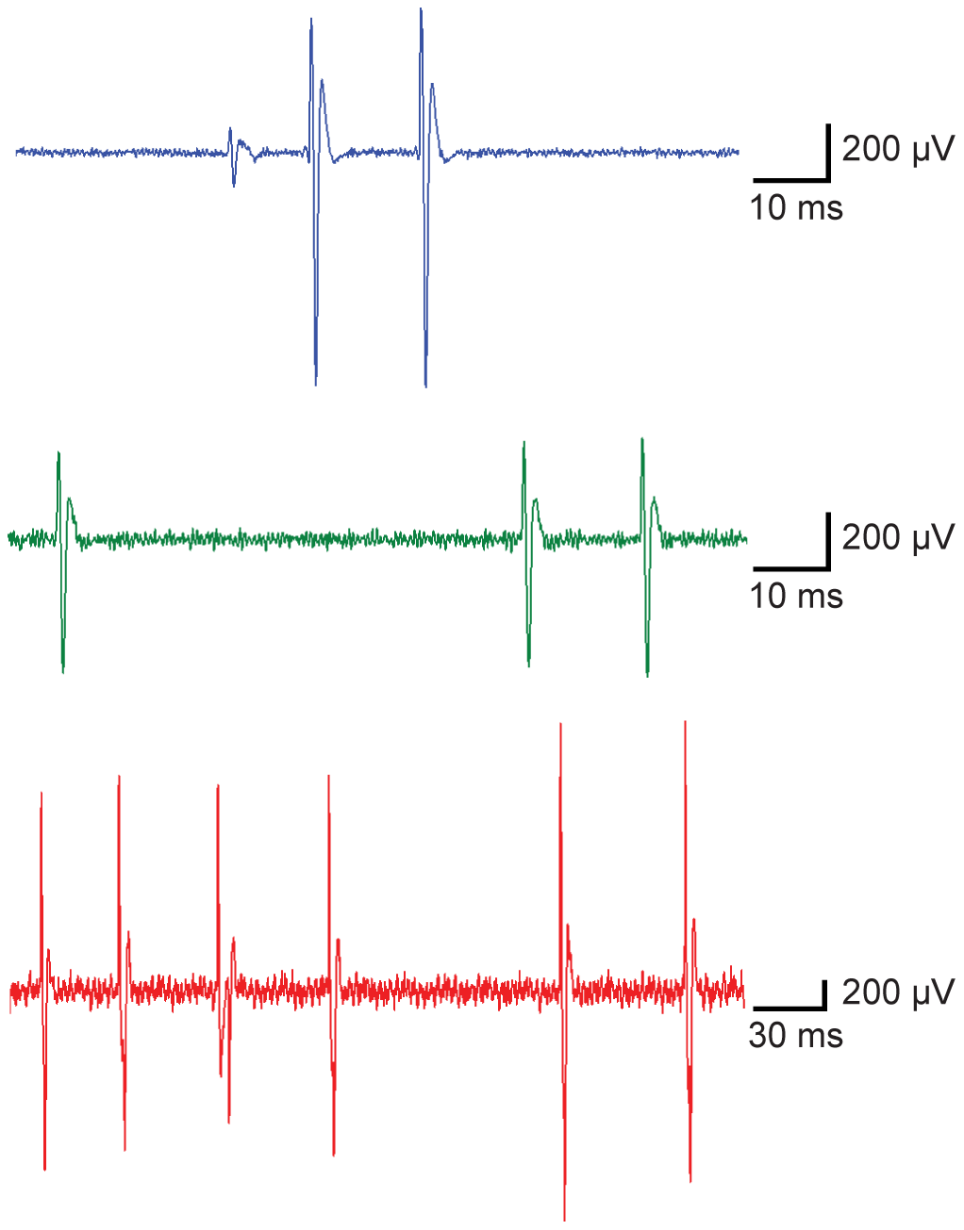
*In vivo* extracellular recordings from mouse brain somatosensory cortex. Three different extracellular recordings show well-isolated single unit activity.

We also explored whether the CNT probe could be used to record neural activity in the somatosensory cortex of the anesthetized mouse (*n* = 2). When advanced into the cortex, all seven CNT probe samples were able to detect single and/or multi-unit extracellular activity, in some cases revealing extremely large amplitude single unit events with large signal to noise ratio (S/N = 9 − 34, Figure 6). Although intracellular recording configurations were not achieved in these preliminary experiments, the successful intracellular recordings made in brain slices suggest that such recordings are feasible using CNT probes *in vivo*. An additional interesting observation was elastic deformation of the CNT probes: when a small amount of off-axis force was applied to the CNT probes brain tissue, the shaft of the probe bent elastically without snapping. Because mechanical resilience of a neural electrode is crucial for *in vivo* intracellular recordings, the elastic feature of the CNT probe could offer advantages over conventional glass electrodes for such an application.

## Discussion

Here we developed a novel CNT probe suitable for intracellular and extracellular recordings from vertebrate neurons. The advantages of CNT probes include lower impedance than glass sharp recording electrodes, mechanical flexibility, and bio-compatibility, suggesting that they may be suitable for a broad range of recording applications *in vivo* and *in vitro*. We could reuse the probes without any treatment as long as we didn’t physically damage the probes by hitting a hard surface or applying excessive off-axis force. Full realization of this potential will require improving insulation layers, implementing capacitance compensation methods, and further refinement of the probe geometry. Nonetheless, the CNT probe developed here presents a new and simple device for use as an interface with neural tissue.

## Supporting Information

Figure S1 **Electrochemical impedance spectroscopy (EIS) of CNT probe with the conformal Parylene-C coating (no FIB).** Left: Impedance over the frequency range of 1 to 100 kHz; Right: Nyquist plot of the same measurement.
(TIF)

Figure S2 **Time evolution of intracellular recording (membrane potential (mV) vs. time (ms)).** The recording is pseudo-continuous by repeating 2.5 second recording and 3.4 second gap. The figures show typical time evolution of a recording (t in each figure is starting time in seconds).
(TIF)

Figure S3 **Action potential time course during intracellular recording.** (A) Magnitude of Action Potentials (mV, peak value – membrane potential). (B) Full width half maximum of action potentials (ms). Data points are sampled over recording period for each cell.
(TIF)

Appendix S1 **Methods: Further description of EIS measurement.**
(DOC)
