# Supplementary figures and images for: Intracellular Neural Recording with Pure Carbon Nanotube Probes
Source: PLoS One. 2013 Jun 19;8(6):e65715. doi: 10.1371/journal.pone.0065715 (PMC3686779; doi:10.1371/journal.pone.0065715)

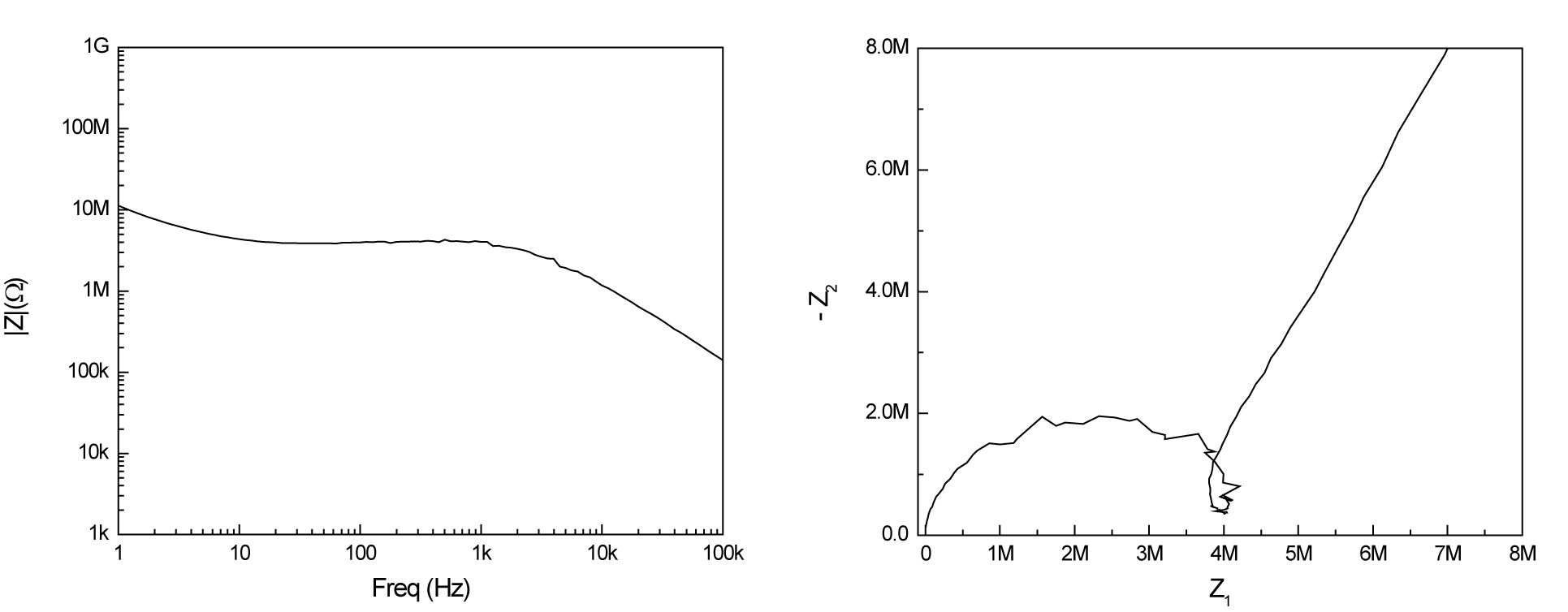

Supplement: Figure S1 — Electrochemical impedance spectroscopy (EIS) of CNT probe with the conformal Parylene-C coating (no FIB). Left: Impedance over the frequency range of 1 to 100 kHz; Right: Nyquist plot of the same measurement. (TIF) [file pone.0065715.s001.tif]

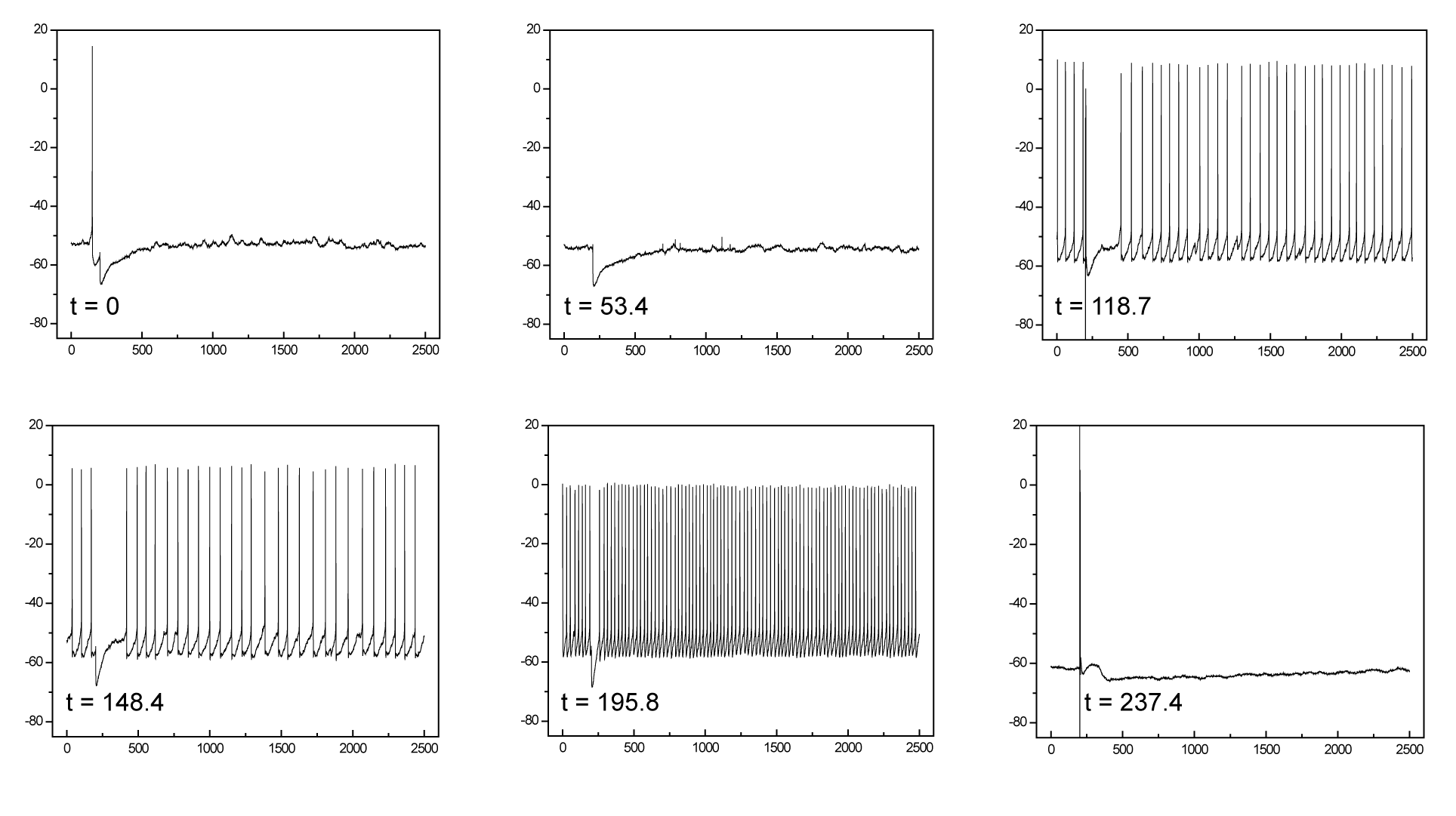

Supplement: Figure S2 — Time evolution of intracellular recording (membrane potential (mV) vs. time (ms)). The recording is pseudo-continuous by repeating 2.5 second recording and 3.4 second gap. The figures show typical time evolution of a recording (t in each figure is starting time in seconds). (TIF) [file pone.0065715.s002.tif]

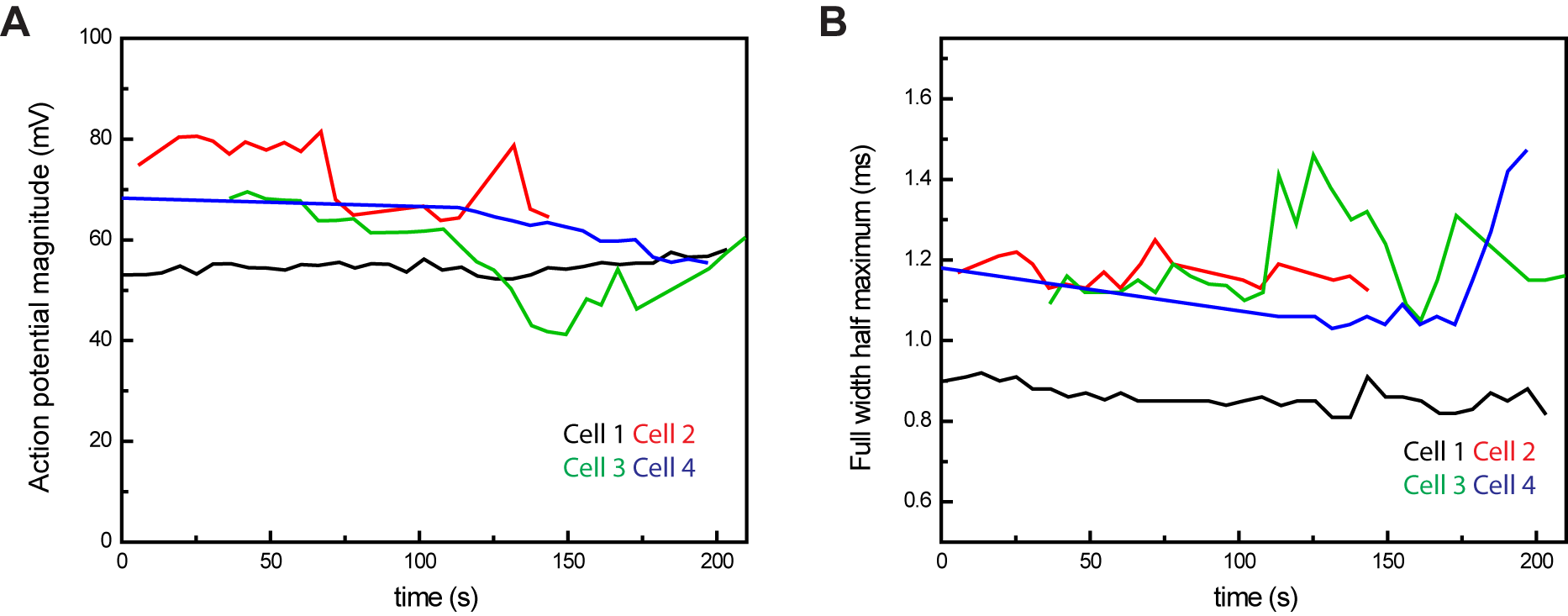

Supplement: Figure S3 — Action potential time course during intracellular recording. (A) Magnitude of Action Potentials (mV, peak value – membrane potential). (B) Full width half maximum of action potentials (ms). Data points are sampled over recording period for each cell. (TIF) [file pone.0065715.s003.tif]
