# Supplementary material for: Intracellular Neural Recording with Pure Carbon Nanotube Probes
Source: PLoS One. 2013 Jun 19;8(6):e65715. doi: 10.1371/journal.pone.0065715 (PMC3686779; doi:10.1371/journal.pone.0065715)
Supplement: Appendix S1 — Methods: Further description of EIS measurement. (DOC) [file pone.0065715.s004.doc]

**Supporting Text Appendix S1 for:**

**Intracellular neural recording with pure carbon nanotube probes**

Inho Yoon^1^, Kosuke Hamaguchi^2^, Ivan V. Borzenets^3†^, Gleb Finkelstein^3^, Richard Mooney^2*^, and Bruce R. Donald^4,5,1*^

^1^Department of Electrical and Computer Engineering, Duke University, Durham, North Carolina, USA.

^2^Department of Neurobiology, Duke University Medical Center, Durham, North Carolina, USA.

^3^Department of Physics, Duke University, Durham, North Carolina, USA.

^4^Department of Computer Science, Duke University, Durham, North Carolina, USA.

^5^Department of Biochemistry, Duke University Medical Center, Durham, North Carolina, USA.

†Present address: Department of Applied Physics, University of Tokyo, Tokyo, Japan.

***Corresponding Authors:**

**Bruce R. Donald: brd+plos13@cs.duke.edu**

P.O. Box 90129 Departments of Computer Science, Biochemistry, and Electrical and Computer Engineering, Duke University, Duke University Medical Center

Durham, NC 27708-0129 USA

Phone: 919-660-6583

**Richard Mooney: mooney@neuro.duke.edu**

P.O. Box 3209, Depart. of Neurobiology, Duke University Medical Center

Durham, NC 27710 USA

Phone: 919-684-5025 Ext.2081

**Methods: Further description of EIS measurement**

As mentioned in the main text, the interface between an ionic solution and CNT is complex to model. In addition, our CNT probes have thin insulation and pores at different scales (micropores (diameter (*d*) < 2 nm), mesopores (2 nm < *d* < 50 nm), and macropores (*d* > 50 nm)) which add more complexity [1]. When the CNT probe touches the PBS solution, the PBS solution wets near the tip area including not only the exposed CNTs’ surface but also the insulation coating. Therefore, any impedance measurement includes capacitance due to the insulation, and increasing the dipping depth amplifies the capacitive component in the measurement.

Figure S1 shows EIS measurement of a CNT probe with conformal Parylene-C coating (no FIB). For this measurement, the dipping depth was around 1 mm. The measurement on left shows magnitude of impedance over 1 Hz to 100 kHz, and the right figure is a Nyquist plot of the same measurement (-Z_2_ (negative imaginary part of impedance) vs. Z_1_ (real part of impedance). From the measurement, the estimated interfacial resistance and Faradaic leakage resistance are 6 kΩ and 4 MΩ respectively (based on an equivalent circuit model [2]).

In related, but different work, Inaba et al. [3] observed a similar trend of impedance vs. frequency for their *single-wall* CNT (with a Parylene-C coating). They explained the trend by stating that plateauing impedance at lower frequency range is due to dominating diffusion impedance for the lower range, while the effect at higher frequency range is largely due to capacitive impedance coming from the insulation.

**References**

1. Pandolfo AG, Hollenkamp AF (2006) Carbon properties and their role in supercapacitors. Journal of Power Sources 157: 11-27.

2. Conway BE (1999) Electrochemical supercapacitors : scientific fundamentals and technological applications. New York: Plenum Press. pp. 554-555.

3. Inaba A, Takei Y, Kan T, Matsumoto K, Shimoyama I (2012) Electrochemical impedance measurement of a carbon nanotube probe electrode. Nanotechnology 23: 485302
